# Lipidated Stapled Peptides Targeting the Acyl Binding Protein UNC119

**DOI:** 10.1002/cbic.201900615

**Published:** 2019-11-19

**Authors:** Philipp M. Cromm, Hélène Adihou, Shobhna Kapoor, Mercedes Vazquez‐Chantada, Paul Davey, David Longmire, Elisabeth Hennes, Walter Hofer, Philipp Küchler, Elisabetta Chiarparin, Herbert Waldmann, Tom N. Grossmann

**Affiliations:** ^1^ Department of Chemical Biology Max-Planck-Institute of Molecular Physiology Otto-Hahn-Strasse 11 44227 Dortmund Germany; ^2^ Technische Universität Dortmund Fakultät für Chemie und Chemische Biologie Otto-Hahn-Strasse 6 44227 Dortmund Germany; ^3^ Early CVRM Medicinal Chemistry R&D BioPharmaceuticals AstraZeneca Pepparedsleden 1 431 83 Mölndal Sweden; ^4^ Chemistry, Oncology R&D AstraZeneca Darwin Building 310 Cambridge Science Park Milton Road Cambridge CB4 0WG UK; ^5^ Vrije Universiteit Amsterdam Department of Chemistry and Pharmaceutical Sciences De Boelelaan 1083 1081 HV Amsterdam The Netherlands; ^6^ Present address: Research and Development Pharmaceuticals Bayer AG Muellerstrasse 178 13353 Berlin Germany

**Keywords:** macrocyclization, peptidomimetics, protein–protein interaction, trafficking, alpha-helixes

## Abstract

The acyl‐binding UNC119 proteins mediate the activation and transport of various N‐myristoylated proteins. In particular, UNC119a plays a crucial role in the completion of cytokinesis. Herein, we report the use of a lipidated peptide originating from the UNC119 binding partner Gnat1 as the basis for the design of lipidated, stabilized α‐helical peptides that target UNC119a. By using the hydrocarbon peptide‐stapling approach, cell‐permeable binders of UNC119a were generated that induced the accumulation of cytokinetic and binucleated cells; this suggests UNC119a as a potential target for the inhibition of cytokinesis.

## Graphical abstract

**Targeting molecular chaperones**: Cell‐permeable peptidomimetics of a myristoylated peptide were obtained by hydrocarbon peptide stapling. These agents target the human acyl‐binding protein UNC119a, which plays a crucial role in cytokinesis, inducing the accumulation of cytokinetic and binucleated cells. This suggests UNC119a as a potential target for the inhibition of cytokinesis.

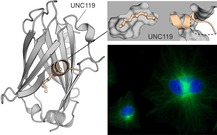

Protein lipidation is a unique post‐translational modification that plays a crucial role in protein localization and function.^1^, ^2^, ^3^ Lipidation increases the chemical and functional complexity of the proteome by the covalent attachment of hydrophobic functionalities allowing soluble proteins to be located to the cell membrane. Many cellular signaling events are dependent on such membrane association, and the correct localization of signaling proteins is crucial for cell function and survival.^1^, ^3^ Thus, the inhibition of protein lipidation has emerged as a potential therapeutic strategy for a variety of human diseases.^4^ Myristoylation represents an important form of lipidation and describes the irreversible attachment of a 14‐carbon fatty acid to the N‐terminal glycine of a protein by *N*‐myristoyl transferases.^3^, ^5^, ^6^ In combination with a second membrane linker or positively charged protein stretches, myristoylation modulates protein–membrane as well as protein–protein interactions thereby controlling the subcellular trafficking of proteins.^3^

Myristoylation‐dependent trafficking is regulated by certain chaperones that shield the fatty acid from solvent exposure and thus facilitate inter‐membrane shuttling. The two acyl binding proteins UNC119a and UNC119b (uncoordinated 119) have been shown to bind myristoylated proteins and mediate their intermembrane transport.^7^, ^8^ UNC119b regulates the delivery of myristoylated cargo to the cilium,^9^, ^10^ whereas UNC119a binds and activates Src‐family kinases and is a key player in the transport of transducing α‐subunits.^11^ UNC119a malfunction has been associated with diseases such as retina degradation and blindness.^12^ SiRNA knockdown of UNC119a interferes with the completion of cytokinesis and results in an increased number of binucleated cells.^13^ The absence of UNC119a prevents the activation and correct localization of Fyn^14^ a proto‐oncogene nonreceptor tyrosine kinase, leading to incomplete cytokinesis. As UNC119b is mainly associated with transport mechanisms in the cilium, whereas UNC119a mediates Src‐family kinase activation and cytokinesis, specific inhibition of UNC119a could be desirable.^15^

UNC119a interacts with a variety of N‐myristoylated proteins and is able to selectively bind to short N‐myristoylated peptides (Figure 1 A), while showing low or no affinity for alternatively lipidated or prenylated peptide sequences.^16^ The co‐crystal structure of UNC119a with an N‐terminal lauroylated (12‐carbon) peptide fragment derived from the transducin α‐subunit Gnat1 revealed that the lipid chain and the first six amino acids are buried within the hydrophobic cavity of UNC119a (Figure 1 B).^8^, ^17^ The N‐terminally lipidated peptide sequence forms a short N‐terminal α‐helix (six amino acids) that is completely inserted into the UNC119a binding pocket, and is accompanied by a three‐residue stretch that adapts an extended conformation and does not show direct contacts with UNC119a (Figure 1 B).^8^ Most previous attempts to impair the spatial and temporal regulation of lipidated proteins targeted their synthesis machinery;^4^ only a few examples are known that target the transport mechanism of lipidated proteins.^18^, ^19^, ^20^, ^21^, ^22^, ^23^ To evaluate the potential of peptide‐derived inhibitors of UNC119a, we designed Gnat1‐derived lipidated stapled peptides. These peptides show nanomolar affinities and cellular activity by hampering cytokinesis and thereby increasing the number of binucleated cells.

**Figure 1:**
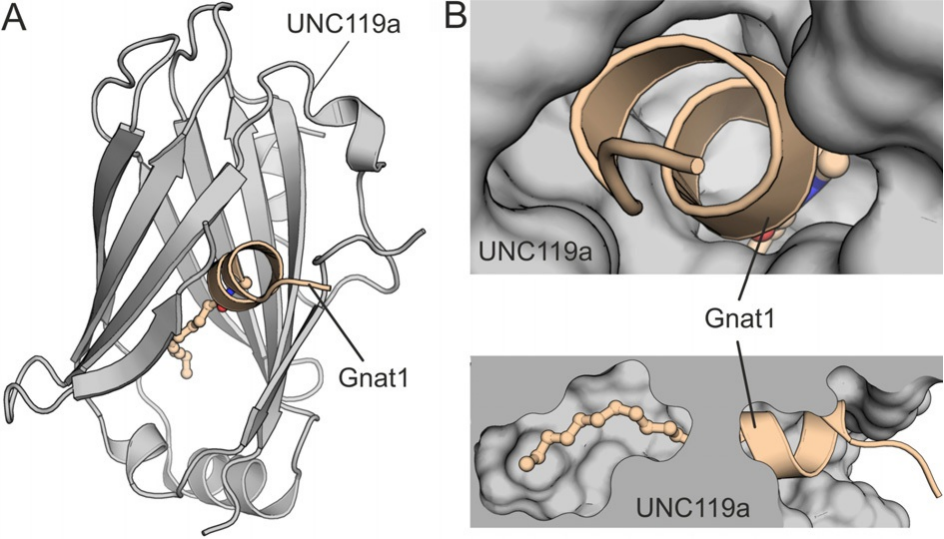
A) Crystal structure of the lauroylated N terminus of Gnat1 bound to UNC119a (PDB ID: 3RBQ); B) The N‐terminal α‐helix is inserted into the lipid binding pocket of UNC119a with only the unordered part of the peptide standing out (PDB ID: 3RBQ).^8^

We considered the N‐terminal peptide of Gnat1, which contains the short α‐helical stretch, as the starting point for our inhibitor design (Figure 2 A). Constrained α‐helical binding epitopes have shown increased binding affinities and cell permeability, and have therefore been used to inhibit a variety of intracellular protein–protein interactions.^24^, ^25^, ^26^ In this respect, the hydrocarbon‐stapling technique has proven particularly successful, providing a number of biologically active inhibitors of protein–protein interactions.^26^, ^27^ We envisaged the design of hydrocarbon‐stapled peptides as inhibitors of UNC119. To evaluate binding affinities, the corresponding Gnat1 peptide fragment (aa 1–11) was obtained by solid‐phase peptide synthesis, subsequently N‐terminally myristoylated and labeled with fluorescein at the C‐terminal lysine side chain (peptide **1**, Figure S9 in the Supporting Information). Affinity measurements from a fluorescence anisotropy assay revealed high binding affinities of **1** to UNC119a and UNC119b (dissociation constants*: K*_d_=7.4 and 11.2 nm, respectively, Figure 2 B).

**Figure 2:**
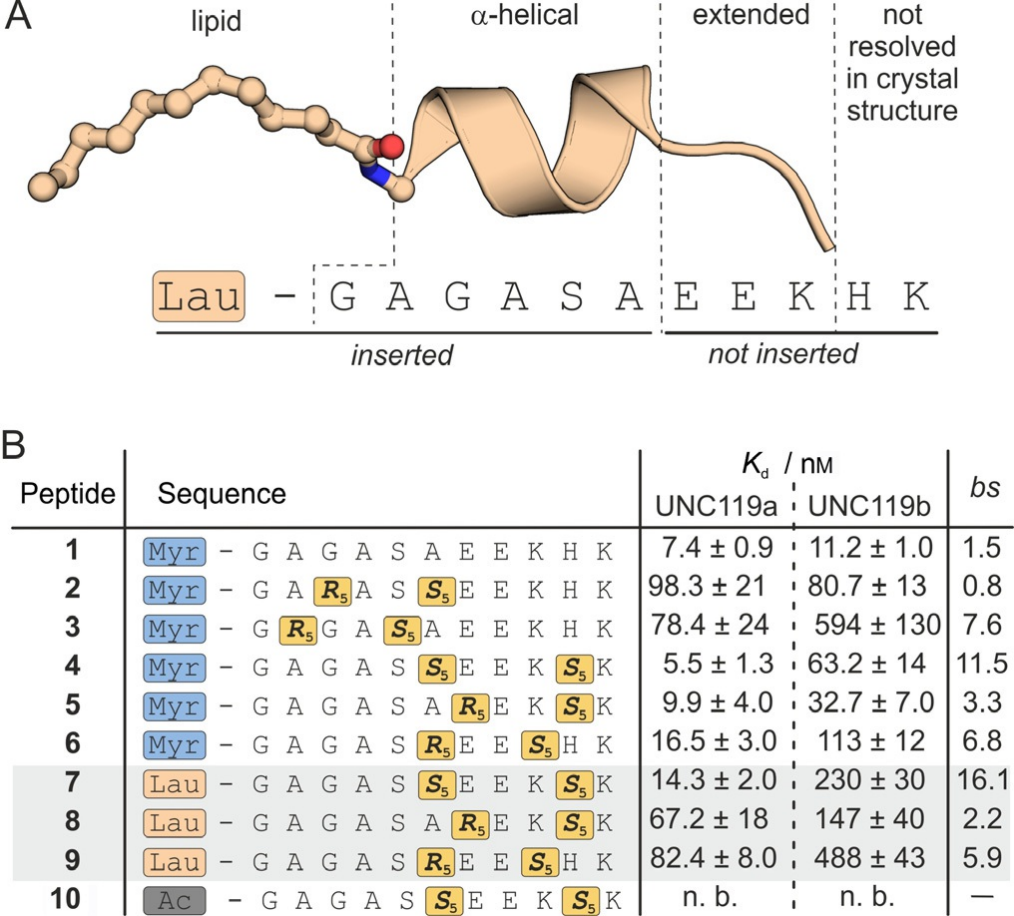
A) Structure and sequence of the lauroylated UNC119a/‐b binding stretch of Gnat1. For clarification, the peptide sequence is divided into various sections (PDB ID: 3RBQ); B) Sequences and binding affinities of the tested UNC119‐binding peptides (**1**–**10**, for details on the peptides, see Figure S1 and Table S1). The α‐methylated building blocks and N‐terminal modifications are highlighted. Binding selectivity: *bs*=*K*_d_[UNC119b]/*K*_d_[UNC119a]; triplicate measurements, errors represent 1 *σ*“; n.b. no binding (for binding curves, see Figure S2).

Stapled peptides based on **1** were designed by placing an *i*,*i*+3 hydrocarbon staple into the α‐helical stretch. The resulting peptides, **2** and **3**, bound UNC119a with nanomolar affinities (*K*_d_=98.3 and 78.4 nm, respectively), but showed about tenfold reduced affinity compared to the parent peptide (Figure 2 B). The N‐terminal α‐helix is buried in the UNC119a binding pocket; presumably this interferes with the sterically demanding residues introduced for crosslinking—in line with previous attempts to crosslink fully buried binding epitopes.^28^, ^29^ Therefore, we moved the hydrocarbon staple towards the C terminus, avoiding potential steric clashes. The resulting myristoylated stapled peptides with either *i*,*i*+4 (peptide **4**) or *i*,*i*+3 (peptide **5**, **6**) crosslinks showed considerably higher binding affinities towards UNC119a than the first‐generation peptides **2** and **3** (Figure 2 B). For UNC119a, **4** and **5** exhibit a dissociation constant (*K*_d_=5.5 and 9.9 nm, respectively) in the range of peptide **1**.

Having a panel of stapled Gnat1‐derived peptides in hand, we assessed their selectivity for binding of the isoforms UNC119a and ‐b. The affinities for UNC119b were determined (Figure 2 B) and revealed that all stapled peptides, except **2**, have an increased binding preference for UNC119a compared to starting peptide **1**. Most notably, **4** (Figure S10), which exhibits the highest affinity for UNC119a, also shows the most pronounced UNC119a/‐b discrimination in our panel (11.5 times higher affinity for UNC119a, Figure 2 B). UNC119a and UNC119b share a sequence homology of 60 %^15^ with a number of variations close to the peptide binding site (Figure S3); this might be the cause for observed changes in binding selectivity. Knowing about the preference of UNC119 for myristorylated peptides, we were interested how a reduction in the fatty acid length affects binding. For this reason, the stapled peptides with the highest affinity (**4**, **5** and **6**) were equipped with lauric acid (12 C atoms) instead of myristic acid (14 C atoms). The corresponding peptides **7**, **8** and **9** showed the expected reduced binding affinity. As one would expect, removal of lipidation thwarts binding completely, as shown for peptide **10**, the acetylated analogue of peptide **4**.

As the knock‐down of UNC119a was reported to increase the number of cytokinetic and binucleated cells,^13^ we investigated the influence of UNC119‐binding peptides on cell morphology. Initially, the uptake of fluorescently labeled peptide **4** by HeLa cells was investigated by using flow cytometry (*c=*5 μm for 90 min, Figure S4 and Table S2). These measurements show cellular uptake of **4** reaching about 50 % of the levels observed for cell‐penetrating peptide octa‐arginine (R_8_).^30^ Considering that this cellular uptake was sufficient, we tested the effect of unlabeled peptide **4** on cell morphology. As a reference, we used nocodazole (NC), which inhibits tubulin polymerization thereby inducing cell‐cycle arrest in the G2/M phase that results in cells with increased DNA content because chromosomal DNA has already been duplicated. HeLa and mouse fibroblast L cells were incubated with different peptide concentrations for 72 h prior to quantification of DNA content by using flow cytometry. Very similar effects were observed for both cell lines (Figure S5), and as expected, NC treatment resulted in increased numbers of cells with doubled DNA content (4 *N*, *N*=number of chromosome sets, Figures 3 A and S6). Most strikingly, treatment with peptide **4** also caused cells to accumulate in the 4 *N* state. At a concentration of 50 μm, 36 % of HeLa cells (Figure S6) and 42 % of L cells (Figure 3 A) showed a DNA content of 4 *N*. Importantly, this effect is dose‐dependent. Parent peptide **1** also showed some effect (at 50 μm, 24 and 28 % of cells with DNA content of 4 *N*), but not as pronounced as that of stapled peptide **4**. Consistent with an UNC119‐dependent effect, nonbinding peptide **10** had no impact on the DNA content in either cell line.

**Figure 3:**
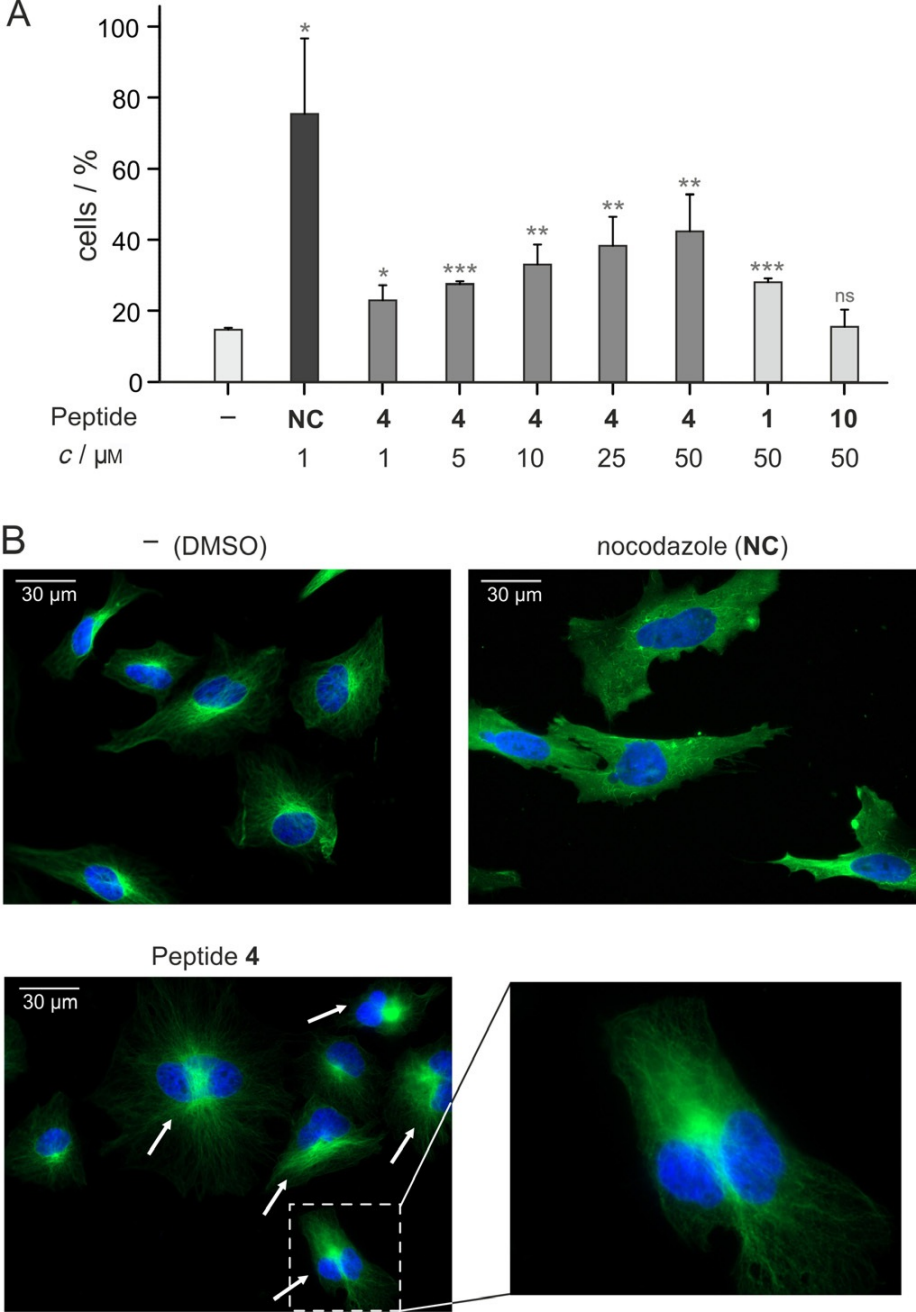
A) Unlabeled peptide **4** increases the number of cells with 4 *N* DNA content (*N*=number of chromosome sets). L cells were treated with peptide for 72 h or not (NC served as the control). The DNA content of L cells was determined by propidium iodide staining and flow cytometry. Results were obtained from triplicate measurements of L cells (errors represent 1 *σ*). Significances compared to DMSO (unpaired t‐test): n.s. *p*>0.05, * *p*<0.05, ** *p*<0.01, *** *p*<0.001. For details, see Figure S5 and Table S2; B) HeLa cell were treated with 25 μm unlabeled peptide **4** for 72 h or 1 μm NC for 24 h prior to fixation and staining of microtubules (green, anti‐tubulin antibody) and DNA (blue, DAPI). Arrows indicate binucleated cells. Magnification shows a binucleated cell after treatment with peptide **4**.

By using microscopy, the effect of peptides on HeLa cell morphology was analyzed after fixation, permeabilization and staining with a tubulin‐selective antibody (green) and DAPI (DNA, blue, Figure 3 B). Compared to untreated cells (DMSO), NC treatment (24 h) resulted in the deformation of the tubulin network. In clear contrast to this behavior, incubation with peptide **4** (72 h) resulted in the formation of binucleated cells. The presence of two nuclei together with the increased number of cells with DNA content of 4 *N* as observed by flow cytometry (Figure 3 A) indicate a failure of cytokinesis. In addition, we note that peptide **4**‐treated cells do not appear to be distorted or stressed, as observed after NC treatment. These observations are line with UNC119 inhibition.

Flow cytometry experiments with fluorescently labeled peptide **4** indicated cellular uptake by HeLa cells (Figure S4). However, these experiments do not allow the subcellular localization of the peptide to be assessed. To better understand the intracellular availability of the peptide, we quantified the peptide in subcellular compartments by using unlabeled peptides, as applied in the morphology studies (Figure 3). The concentration of free compound in subcellular compartments can be measured by quantitative mass spectrometry.^31^, ^32^ We extended this approach, only used for small molecules so far, to peptides, and we measured intracellular concentrations in the nuclear and the cytosolic compartments.^31^, ^33^ Myristoylated stapled **4** and acetylated control **10** along with cell‐penetrating R_8_ were selected for these studies. After incubation for 30 min (*c=*25 μm), MDA‐MB‐231 cells were lysed, and the cell content was reconstituted. The total peptide concentration was then determined by quantitative mass spectrometry. As expected, **10** shows poor cellular uptake (*c=*0.24 pmol per 10^6^ cells, Table 1), whereas **4** and R_8_ show a more than 100‐fold higher cell uptake (*c=*60 and 51.7 pmol per 10^6^ cells, respectively). We then determined peptide concentrations after 0.5 and 72 hours′ incubation in the cytoplasmic and nuclear fractions. After 0.5 h, **10** could not be detected, whereas R_8_ was distributed equally in both compartments (*c*(nucleus)=0.56 and *c*(cytoplasm)=0.58 pmol per 10^6^ cells). On the other hand, **4** accumulates in the nucleus (*c*(nucleus)=0.22 pmol/million cells). Similar cellular uptake is observed after a prolonged incubation (72 h). Overall, these results demonstrate the intracellular availability of peptide **4**, though with pronounced nuclear localization.

**Table 1:** Subcellular distribution of unlabeled peptides investigated by mass spectrometry (0.5 and 72 h incubation at *c*(peptide)=25 μm). If no peptide was detected, the lower limit of quantification is given (for details see Table S7).

	Peptide concentration [pmol per 10^6^ cells]				
Peptide	Total cell	Nucleus		Cytosol	
	0.5 h	0.5 h	72 h	0.5 h	72 h
**4**	60	0.22	0.45	<0.001	0.01
**10**	0.24	<0.04	<0.04	<0.04	<0.04
R_8_	52	0.56	0.64	0.58	0.65

In conclusion, hydrocarbon stapled peptides with increased binding affinity for UNC119a were designed based on the crystal structure of UNC119a and Gnat1. These peptides inhibit UNC119a, a regulator of N‐myristoylated proteins and crucial player in the regulation of cytokinesis. Peptide **4** exhibits a more than 11‐fold higher affinity for UNC119a compared to UNC119b, whereas the wild‐type peptide **1** binds to both isoforms with similar binding affinity. Given the cellular functions of UNC119a and UNC119b, selective targeting of UNC119a is most likely desirable due to UNC119a′s involvement in cytokinesis and Src‐family kinase activation. Cellular treatment with UNC119a inhibitor **4** results in peptide accumulation in the nuclear compartment as well as a significantly increased number of cells with doubled DNA content. Microscopy studies link the increase in DNA content to the formation of binucleated cells, thus indicating inhibition of cytokinesis. This resembles a mode of action different from that of the tubulin inhibitor nocodazole. These results are in agreement with previous reports showing an increased accumulation of binucleated HeLa cells and thus impaired cytokinesis upon UNC119a knockdown.^13^ Our findings verify UNC119a as target for cytokinesis inhibition and encourage further optimization efforts^34^, ^35^ towards stable and bioavailable peptidomimetics.

## Supporting information

As a service to our authors and readers, this journal provides supporting information supplied by the authors. Such materials are peer reviewed and may be re‐organized for online delivery, but are not copy‐edited or typeset. Technical support issues arising from supporting information (other than missing files) should be addressed to the authors.

Supplementary
