# Supplementary material for: Lipidated Stapled Peptides Targeting the Acyl Binding Protein UNC119
Source: Chembiochem. 2019 Nov 19;20(24):2987–90. doi: 10.1002/cbic.201900615 (PMC6973269; doi:10.1002/cbic.201900615)
Supplement: Supplementary file 1 — Supplementary [file CBIC-20-2987-s001.pdf]

CHEMBIOCHEM

Supporting Information

Lipidated Stapled Peptides Targeting the Acyl Binding Protein UNC119

Philipp M. Cromm,[a, b, f] Hélène Adihou,[a, c] Shobhna Kapoor,[a] Mercedes Vazquez-Chantada,[d] Paul Davey,[d] David Longmire,[d] Elisabeth Hennes,[a] Walter Hofer,[a] Philipp Küchler,[a] Elisabetta Chiarparin,[d] Herbert Waldmann,*[a, b] and Tom N. Grossmann*[e]

cbic_201900615_sm_miscellaneous_information.pdf

Supporting Information

Lipidated stapled peptides targeting the acyl binding protein UNC119

Philipp M. Cromm[1,2#], Hélène Adihou[1,3], Shobhna Kapoor[1], Mercedes Vazquez-Chantada[4], Paul Davey[4], David Longmire[4], Elisabeth Hennes[1], Walter Hofer[1], Philipp Küchler[1], Elisabetta Chiarparin[4], Herbert Waldmann[1,2,], Tom N. Grossmann[5]**

[1] Department of Chemical Biology, Max-Planck-Institute of Molecular Physiology, Otto-Hahn-Strasse 11, D-44227 Dortmund, Germany

[2] Technische Universität Dortmund, Fakultät für Chemie und Chemische Biologie, Otto-Hahn-Strasse 6, D-44227 Dortmund, Germany

[3] Medicinal Chemistry, Research and Early Development Cardiovascular, Renal and Metabolism, BioPharmaceuticals R&D, AstraZeneca, Gothenburg, Sweden;

[4] Chemistry, R&D Oncology , AstraZeneca , Cambridge CB4 0QA , United Kingdom

[5] VU University Amsterdam, Department of Chemistry & Pharmaceutical Sciences, De Boelelaan 1083, 1081 HV, Amsterdam, The Netherlands

[#]Current address: Research and Development, Pharmaceuticals, Bayer AG, 13353 Berlin, Germany

* To whom correspondence should be addressed. Email: t.n.grossmann@vu.nl, herbert.waldmann@mpi-dortmund.mpg.de

Table of contents

1 Chemical Methods 2

2 Biochemical Methods 5

3 Supporting Figures 8

4 Supporting Tables 18

5 References 25

1 Chemical Methods

Chemicals and instrumentation

Unless otherwise noted, chemicals were purchased from Sigma Aldrich, Merck, Okeanos, Roth or Alfa Aesar and were used without further purification. Protected Fmoc-amino acids and coupling reagents were purchased from Novabiochem and Iris Biotech GmbH. Building blocks S_5 and R_5 for hydrocarbon peptide stapling was purchased from Okeanos Tech. Co. LTD. All solvents were purchased from commercial suppliers and used without further purification. Analytical HPLC was performed using an Agilent 1100 Series with either a C18 HPLC column 3 µm (Macherey Nagel) or a C18 HPLC column 1.8 µm (Macherey Nagel). The system was run at a flow rate of 1.0 mL/min over 30 min using H_2O (0.1% TFA) and MeCN (0.1% TFA) as solvents. Linear gradients were run over varying periods of time. HPLC-MS analyses were performed with an Agilent 1100 Series connected to a Thermo LCQ Advantage mass spectrometer using a C18 HPLC column 3 µm (Macherey Nagel). The system was run at a flow rate of 1 mL/min over 15 min using H_2O (0.1% formic acid) and MeCN (0.1% formic acid) as eluents. Semi-preparative HPLC was carried out on a Agilent 1100 Series using a SP125/10 Nuclear C18 Gravity 5 µm column (Macherey Nagel) at a flow rate of 6 mL/min. Linear gradients using H_2O (0.1% TFA) and MeCN (0.1% TFA) were run over varying periods of time. High resolution mass spectra were recorded on a QLT Orbitrap mass spectrometer coupled to an Acceka HPLC-System (HPLC column: Hypersyl GOLD, 50 mm x 1mm particle size 1.9 µm, ionization method: Electrospray Ionization). Automated peptide synthesis was performed using a CEM-Discover microwave and a CEM-Liberty peptide synthesizer. Fluorescence polarization was measured with a Tecan Safire^2. Absorbance measurements were performed on a Tecan infinite M200 and Thermo scientific Nanodrop 2000c. For ITC measurements an iTC_{200} microcalorimeter by MicroCal was used. Data was analyzed using the software Microcal LLC ITC provided by the manufacturer. FACS analysis was performed on a BD LSRII analyzer (Becton Dickinson, USA). Microscope images were obtained on an Axiovert Observer Z1 (Carl Zeiss, Germany).

Peptide synthesis

General

Peptides were synthesized on solid-phase using the Fmoc-strategy and Rink Amide (MBHA) resin, Rink Amide NovaSyn TGR resin or ChemMatrix Rink Amide resin as solid support. Solvents and soluble reagents were removed by suction. Washings between coupling and deprotection were carried out in DMF and DCM using 1 mL solvent per 100 mg resin. Coupling efficiency was monitored by ESI-MS and/or HPLC analyses.

Fmoc deprotection

The resin was swollen in DMF and treated with a solution of piperidine/DMF (20/80, v/v) for 2x 5 min. Afterwards the resin was washed with DMF (3x), DCM (3x) and DMF (3x).

Amino acid coupling/ fatty acid coupling

Fmoc-Xaa-OH (4 eq.) was dissolved in freshly prepared solution of HCTU (3.9 eq., 0.5 M) with DIEA (8 eq.). Subsequently, this mixture was added to the resin and shaken for 30 min at room temperature. For coupling of the alkyne building blocks **1** – **5**, the building blocks **12** – **14** and the subsequent amino acid: Fmoc-Xaa-OH (4 eq.) was dissolved in DMF in the presence of COMU (3.9 eq.), Oxyma (3.9 eq.) and DIEA (8 eq.), added to the resin and shaken for 1 h at room temperature. Except coupling of the alkyne building blocks **1** – **5** and the alkene building blocks **12** – **14**, all couplings were performed as double couplings. All equivalents are calculated based on theoretical loading of the resin as provided by the vendor.

N-terminal acetylation

For preparation of N-acetylated peptides and whenever a quantitative yield even after recoupling treatments was not achieved, the free N-terminal amino group was acetylated using a solution of Ac_2O/DIEA/DMF (1/1/8, v/v/v) for 2x 10 min at room temperature.

Fluorescence labelling with FITC

Prior to fluorescence labelling with FITC the Mtt protecting group of the C-terminal Lys was removed with DCM/TFA/TIPS/Water (994/1/2.5/2.5 v/v/v/v). Afterwards the resin was treated with FITC (5 eq.) and DIEA (10 eq.) for 16 h at room temperature under exclusion of light and the resin was washed with DMF (3x), DCM (3x) and dried to constant weight *in vacuo*.

Ring-closing olefin metathesis

The dried resin was swollen in DCE for 15 min. A solution of Grubbs 1st generation catalyst (2 mg·mL^{-1}) in DCE was added to the resin and reacted for 2 h at room temperature. During the reaction time argon was bubbled through the reaction mixture to remove ethene. The procedure was repeated twice and the resin was washed with DCE (3x), DCM (3x), DMF (3x).

Cleavage from the resin

The dry resin was treated with a solution of TFA/EDT/TIS/H_2O (94/1/2.5/2.5, v/v/v/v) 100 µL for 10 mg resin for 2x 1 h and 1x 5 min. The solvents were evaporated and the crude peptide was precipitated by the addition of cold diethyl ether. After centrifugation (10 min, 16.100 x *g*, 4°C) the supernatant was removed. The crude product was dissolved in H_2O/MeCN (2/1, v/v) and lyophilized. The crude peptides were purified by semi-preparative HPLC.

Fmoc quantification

A defined amount of dry resin was transferred into an Eppendorf cap and treated with 0.5 mL deprotection solution for 15 min. The UV absorption of the supernatant was determined at 305 nm and the occupation density calculated using Beer-Lambert law (ε = 7800 $cm^{-1}\cdot M^{-1}$).

Peptide quantification

The concentration of fluorescein labelled peptides was determined by UV absorption in 20 mM phosphate buffer (pH 8.5) at 496 nm (ε = 77.000 $cm^{-1}\cdot M^{-1}$). The concentration of acetylated peptides was determined gravimetrically or via UV absorption at 280 nm.

2 Biochemical Methods

Fluorescence anisotropy assay

UNC119a/b was serially diluted in a buffer containing 30 mM Tris (pH 7.5), 100 mM NaCl, 1 mM TCEP and 0.01% Chaps (assay buffer), treated with 5 nM fluorescein-labeled peptides and incubated for 4 h at room temperature. Binding assays were performed in 384-well, small volume, black flat-bottom, non-binding plates (Greiner). Fluorescence polarization values (λ_{ex} = 470 nm, λ_{em} = 525 nm) were determined using a Safire II plate reader (Tecan) at room temperature. All measurements were performed in triplicates. After correction for changes in fluorescence intensity upon binding, the fluorescence anisotropy data were converted into fraction bound (equation 1) of the FITC- labeled peptide and fitted via non-linear regression in Prism 5.0 (Graphpad).[1]

$$\text{fraction bound} = \frac{\mathrm{A}-\mathrm{A}_{\mathrm{free}}}{\mathrm{A}-\mathrm{A}_{\mathrm{free}}+\mathrm{Q}(\mathrm{A}_{\mathrm{bound}}-\mathrm{A})} \quad (1)$$

A: observed anisotropy;

A_{free}: anisotropy of free fluorophore

A_{bound}: anisotropy of bound fluorophore

Q: change in fluorescence intensity between free and bound state

Protein expression and purification

UNC119a and UNC119b were expressed in E. coli according to the previously reported procedures.[2]

Cell culture

Mouse L cells and human cervical carcinoma cell line HeLa were obtained from ATCC, USA (ATCC CRL-2468, ATCC CRM-CCL-2). Cells were grown in Dulbecco's Modified Eagle's medium DMEM (PAN Biotech, Germany) supplemented with 10% fetal bovine serum (FBS, Gibc,), 1 mM sodium pyruvate and non-essential amino acids at 37°C and 5% CO_2 in a humidified incubator. Additionally, HeLa cells were supplemented with penicillin and streptomycin. All cultures were routinely tested and found to be free of mycoplasma contamination.

Flow cytometry

3 x10^4 L or Hela cells were seeded per well in 6–well plates and grown overnight. The following day, cells were treated with the indicated compound concentrations for 72 h or with nocodazole as a control for 24 h). Cells were washed, detached using trypsin and re-suspended in phosphate-buffered saline (PBS). Cells were centrifuged at 200xg for 5 min at room temperature (RT) and washed again. Cells were then fixed with 70% ethanol and stained with propidium iodide solution (12 µg/mL propidium iodide (Sigma, Germany), 0.1% (v/v) Triton X-100 and 100 µg/mL DNase-free RNase A (Sigma, Germany) in PBS) for 30 min at room temperature. Cell suspensions were filtered to FACS tubes through a nylon mesh before analysis. 10,000 cells for each sample were sorted by the BD LSRII analyzer (Becton Dickinson, USA). FlowJo 6.2.1 software was used for quantification and analysis of all data. For every analysis, FSC and SSC (forward and side scatter, respectively) gating was performed to exclude doublets and debris. In addition, cells were gated for singlets (PI-W vs. PI-A).

Fluorescence Microscopy

2x10^4 Hela cells were seeded per well in a 24-well plate on cover slips and incubated overnight. Next day, cells were treated with different concentrations of the peptides or DMSO, nocodazole and cytochalasin D as controls and incubated at 37°C and 5% CO_2 for the indicated times (72 h or 24 h). Cells were then fixed using 3.7% paraformaldehyde for 15 min at room temperature followed by permeabilization using 0.1% Triton X-100 in PBS for 15 min. Blocking was performed in 2% BSA in PBS containing 0.1% Tween 20 for 1 h at room temperature. Samples were incubated with the indicated primary antibodies (anti-tubulin primary antibody conjugated to FITC) in the blocking solution overnight at 4°C. After washing, cells were incubated with 1 µg/mL DAPI in blocking solution for 1 h at room temperature, followed by three washes with PBS. Coverslips were then mounted on glass slides and stored at 4°C. Images were acquired on an Axiovert Observer Z1 (Carl Zeiss, Germany) using 63X oil objective and analyzed using ZEN 2011 software (Carl Zeiss, Germany).

Total intracellular and sub cellular concentrations

MDA-MB-321 cells were cultured DMEM (high glucose) supplemented with 10% FCS and 1% Glutamax. When 80% confluency cell culture media was removed from each flask and new media containing 25 µM of peptides was added and incubated with cells for 30 min and 72 h. Following peptide incubation, cells were washed twice with PBS, trypsinized and pelleted. Pellets where lysed in RIPA buffer (Perkin Elmer) for total

lysis; or CelLytic NuCLEAR Extraction Kit (SIGMA) for subcellular fractionation. Total protein quantification BCA (Pierce) was performed prior to MS analysis to normalize sample data.

Total and sub-cellular quantities of peptides **4**, **10** and **R8** were quantified[3] in lysates by UPLC-MS utilising a Waters Xevo TQ-XS (WBA0259) and a Waters Acquity UPLC system consisting of, Sample Manager (M16UFL953M), Acquity PDA (F17UPD457A), Column Oven (E17CMP703G) and Binary Solvent Manager (E17BUR621G). For peptides **4** and **10** analytical UPLC separation was performed using a Waters CSH C18 1.7 µm, 2.1 x 50 mm column. A gradient was utilized of 97% A to 5% in 4mins, 0.5 min hold, 5% A to 97% in 0.5mins, where A = 0.1% HCOOH (aq) and B = 0.1% HCOOH in MeCN at a flow rate of 0.75 mL/min (Supporting Figure S7). Compound **R8** was analyzed in HILIC mode using a Waters BEH Amide 1.7 µm, 2.1 x 100 mm column with a gradient of 30% A to 95% in 1.5 mins, 1 min hold, 95% A to 30% in 1.5 min, 1 min hold at 0.65 mL/min. The Waters TQ-XS was operated in +ve ion Electrospray (ESI) mode with the optimized transitions for PC658-AC, PC658-MYR and Ac-R8 (Supporting Table S3 and Figure S8). The chromatograms at each transition were extracted, smoothed and integrated to give the standard curve. Chromatograms of the samples were treated similarly and by linear regression (1/x) an in-cell concentration was established in the re-suspended cell lysate using the Waters MassLynx TargetLynx™ product (Supporting Tables S4 – S7).

3 Supporting Figures

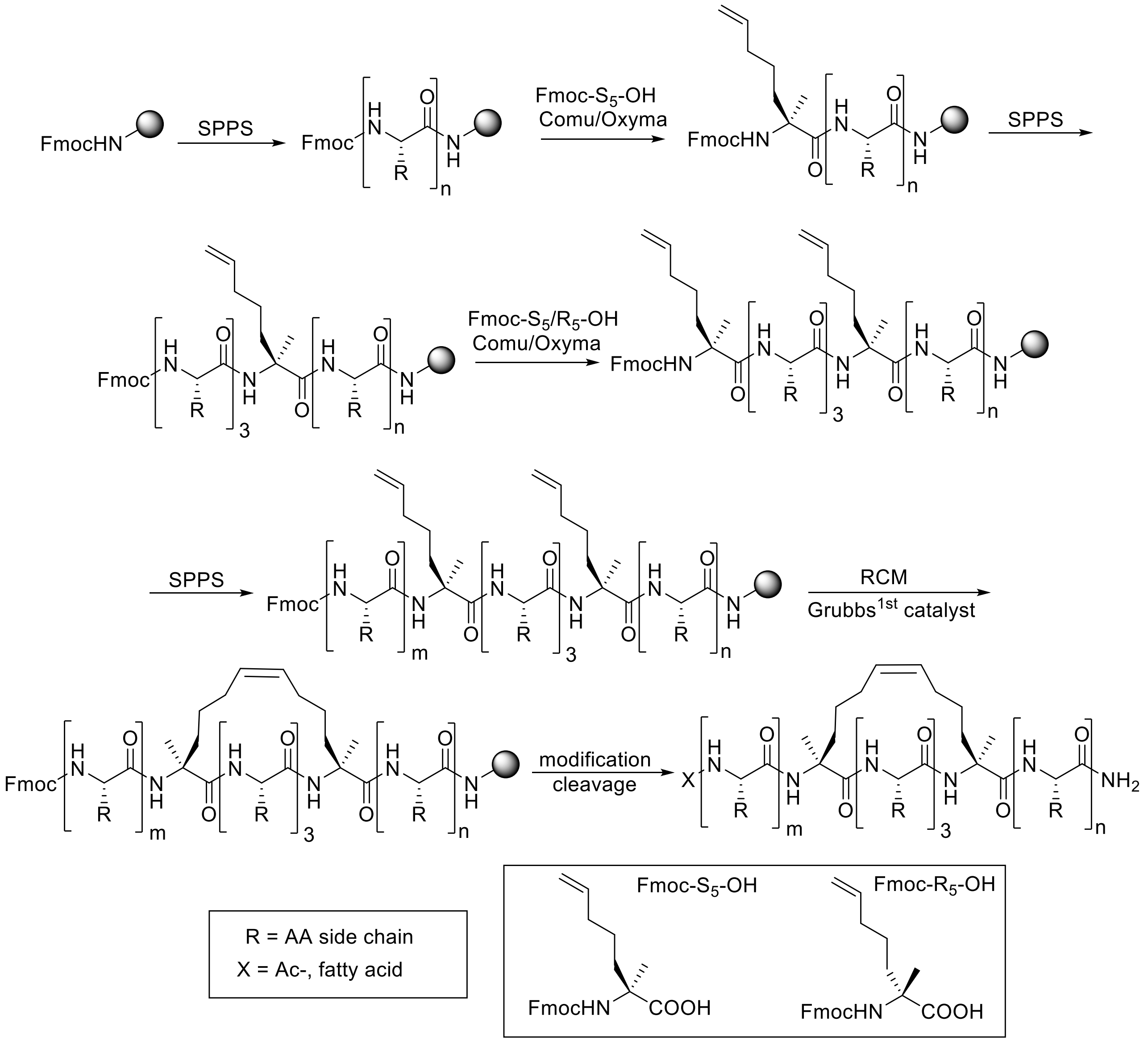

Supporting Figure S1. Synthesis of stapled peptides. A detailed overview of all synthesized peptides (sequences, analytical data) is shown in Supporting Table S1 and Supporting Figures S9 and S10.

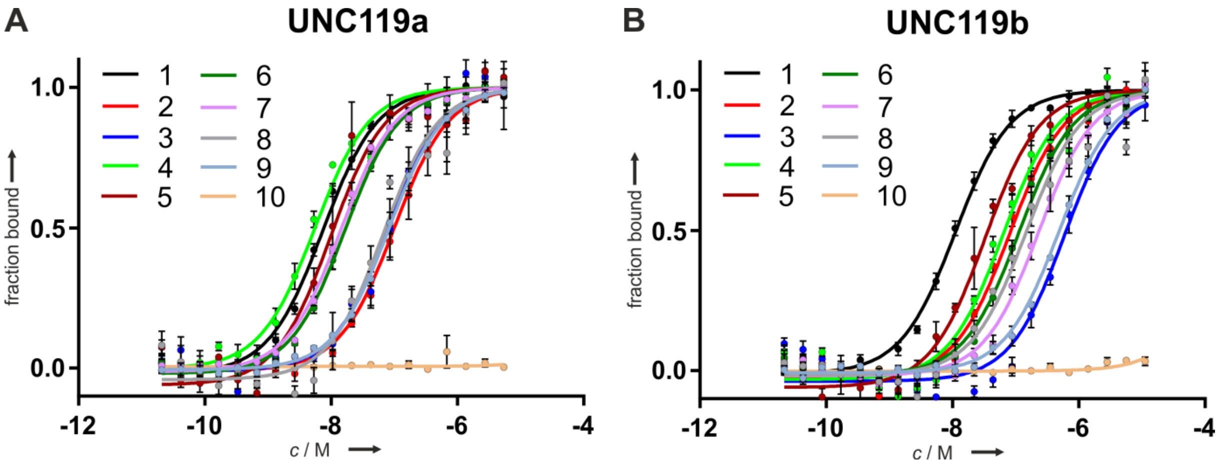

Supporting Figure S2. FP binding graphs of UNC119a (**A**) and UNC119b (**B**).

A

```
sp|Q13432|U119A_HUMAN      MKVKKGGGGAGTATESAPGPSGQSVAPIPQPPAES-----------ESGS
sp|A6NIH7|U119B_HUMAN      ---MSGSNPKAAAAASAAGPGGLVAGKEEKKKAGGGVLNRLKARRQAPHH
                               .*..  .:*: **.**.*  ..   :  * .            .

sp|Q13432|U119A_HUMAN      ESEPDAGPGPRPGPLQRKQPIGPEDVLGLQRITGDYLCSPEENIYKIDFV
sp|A6NIH7|U119B_HUMAN      AADDGVGAAVTEQELLALDTIRPEHVLRLSRVTENYLCKPEDNIYSIDFT
                            :: ..*..     *   :.* **.** *.*:* :***.**:***.***.

sp|Q13432|U119A_HUMAN      RFKIRDMDSGTVLFEIKKPPVSERLPINRR---DLDPNAGRFVRYQFTPA
sp|A6NIH7|U119B_HUMAN      RFKIRDLETGTVLFEIAKPCVSDQEEDEEEGGGDVDISAGRFVRYQFTPA
                           ******:::******* ** **::    :..  *:* .************

sp|Q13432|U119A_HUMAN      FLRLRQVGATVEFTVGDKPVNNFRMIERHYFRNQLLKSFDFHFGFCIPSS
sp|A6NIH7|U119B_HUMAN      FLRLRTVGATVEFTVGDKPVSNFRMIERHYFREHLLKNFDFDFGFCIPSS
                           ***** **************.***********::***.***.********

sp|Q13432|U119A_HUMAN      KNTCEHIYDFPPLSEELISEMIRHPYETQSDSFYFVDDRLVMHNKADYSY
sp|A6NIH7|U119B_HUMAN      RNTCEHIYEFPQLSEDVIRLMIENPYETRSDSFYFVDNKLIMHNKADYAY
                           :*******:** ***::*  **.:****:********::*:*******:*

sp|Q13432|U119A_HUMAN      SGTP
sp|A6NIH7|U119B_HUMAN      NGGQ
                           .*
```

B

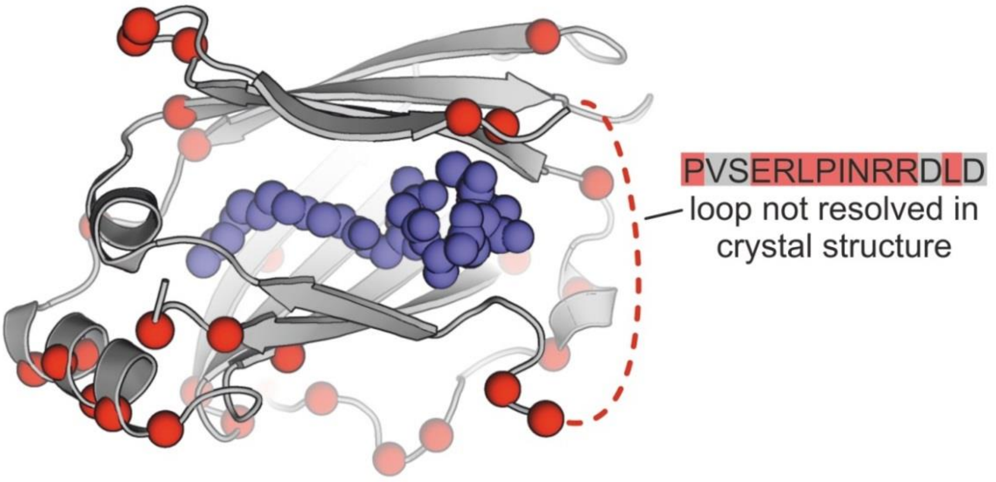

Supporting Figure S3. (**A**) Sequence alignment of UNC119a and UNC119b highlighting the part covered by the crystal structure of UNC119a (PDB 3RBQ) in grey. Variant amino acids are highlighted red. (**B**) Crystal structure (PDB 3RBQ) of UNC119a (grey) in complex with the Gnat1 peptide (blue). Variant positions compared to UNC119b are highlighted red. A very variable loop close to the peptide binding site is not resolved (PVSERLPINRRDLD).

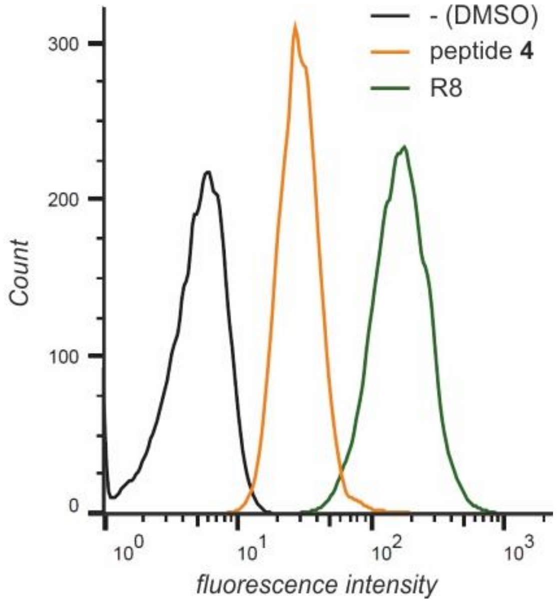

Supporting Figure S4. Cellular uptake of fluorescently labeled peptides as determined by fluorescence intensities using flow cytometry in HeLa cells (90 min incubation at c(peptide) = 5 µM).

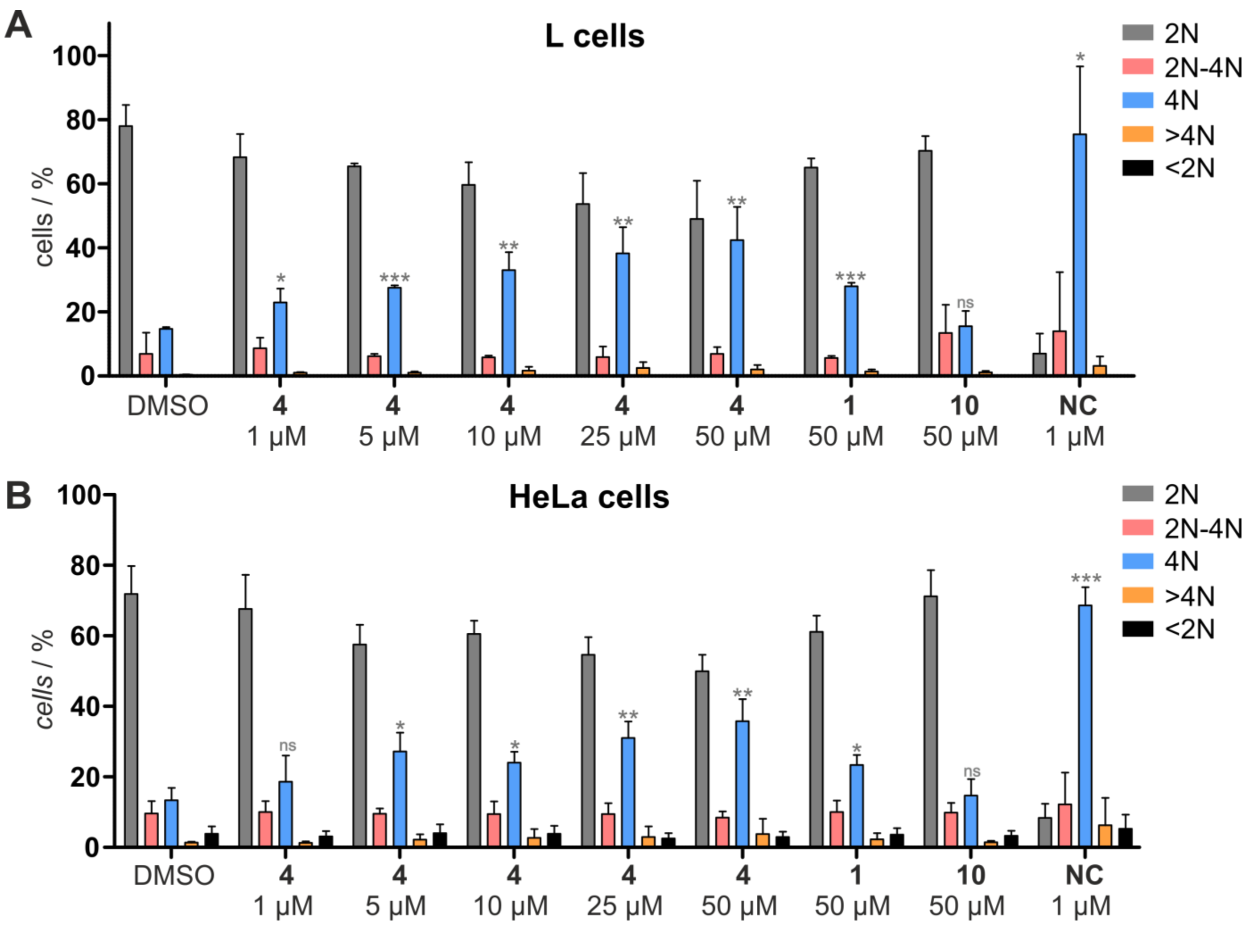

Supporting Figure S5. Influence of peptide treatment on the DNA content. Flow cytometry measurements of peptide **1**, **4** and **10** in mouse fibroblast L cells (**A**) and HeLa cells (**B**). DNA content of L cells and HeLa cells was determined by means of propidium iodide staining and flow cytometry (N = number of chromosome sets).

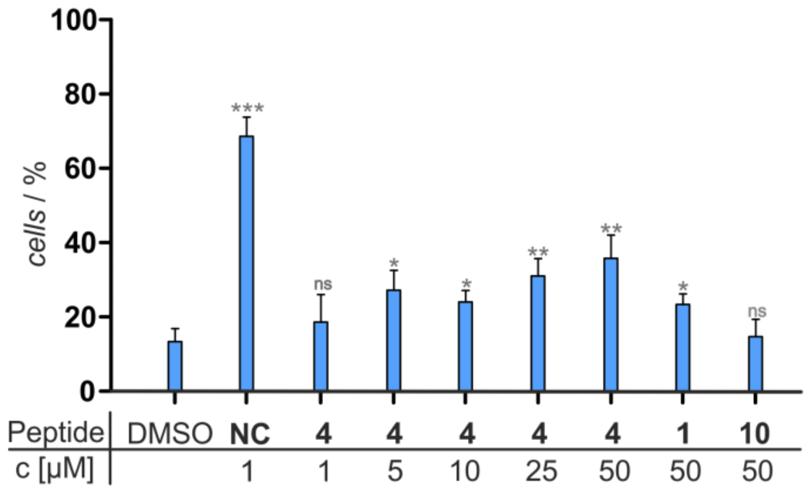

Supporting Figure S6. Influence of peptide treatment on the DNA content. Peptide **4** increases the number of cells with 4N DNA content. HeLa cells were treated with the compounds or DMSO and 1 µM nocodazole as controls for 72 h (nocodazole 24 h). DNA content of HeLa-cells was determined by means of propidium iodide staining and flow cytometry. (errors represent 1 σ). Significance compared to DMSO (unpaired t-test): ns p >0.05, * p <0.05, ** p <0.01, *** p <0.001 (N = number of chromosome sets, for values see Supplementary Table S2).

A **10**: Retention time 0.83mins, S/N 40:1

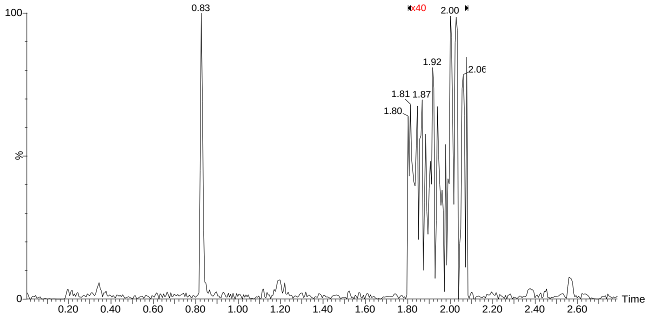

B **4**: Retention time 1.82mins, S/N 500:1

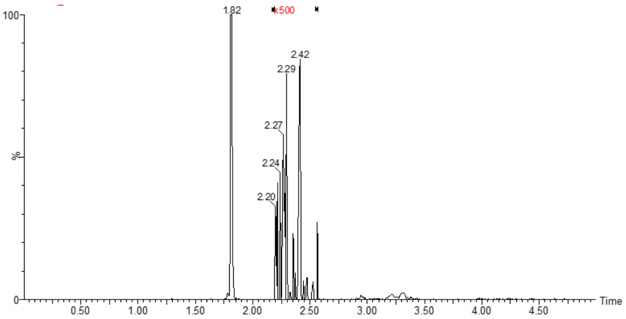

C **R8**: Retention Time 1.14mins, S/N 24:1

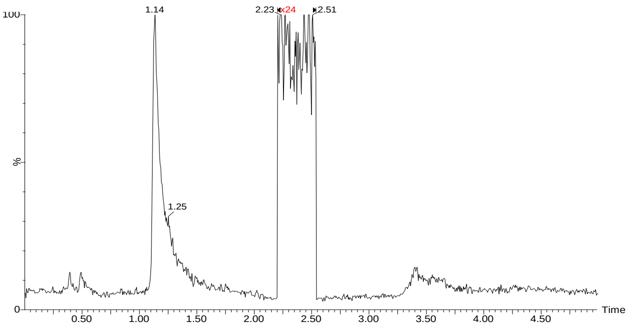

Supporting Figure S7. Representative chromatograms used to determine subcellular concentrations of peptides showing S/N ratio at 50 nM of peptide (**A**) **10**, (**B**) **4** and (**C**) **R8**.

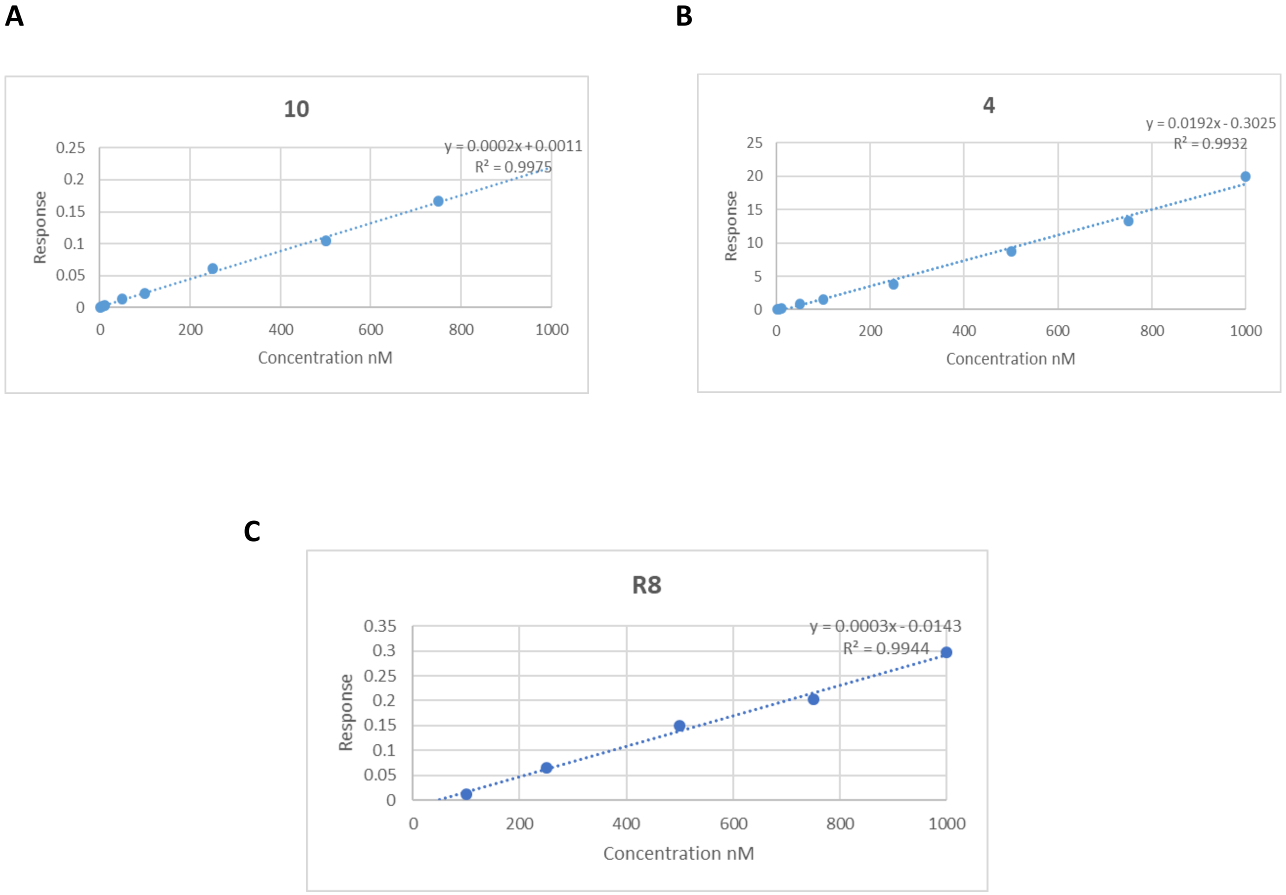

Supporting Figure S8. Representative standard calibration curve of (**A**) **10**, (**B**) **4** and (**C**) **R8** used to determine subcellular concentrations of peptides.

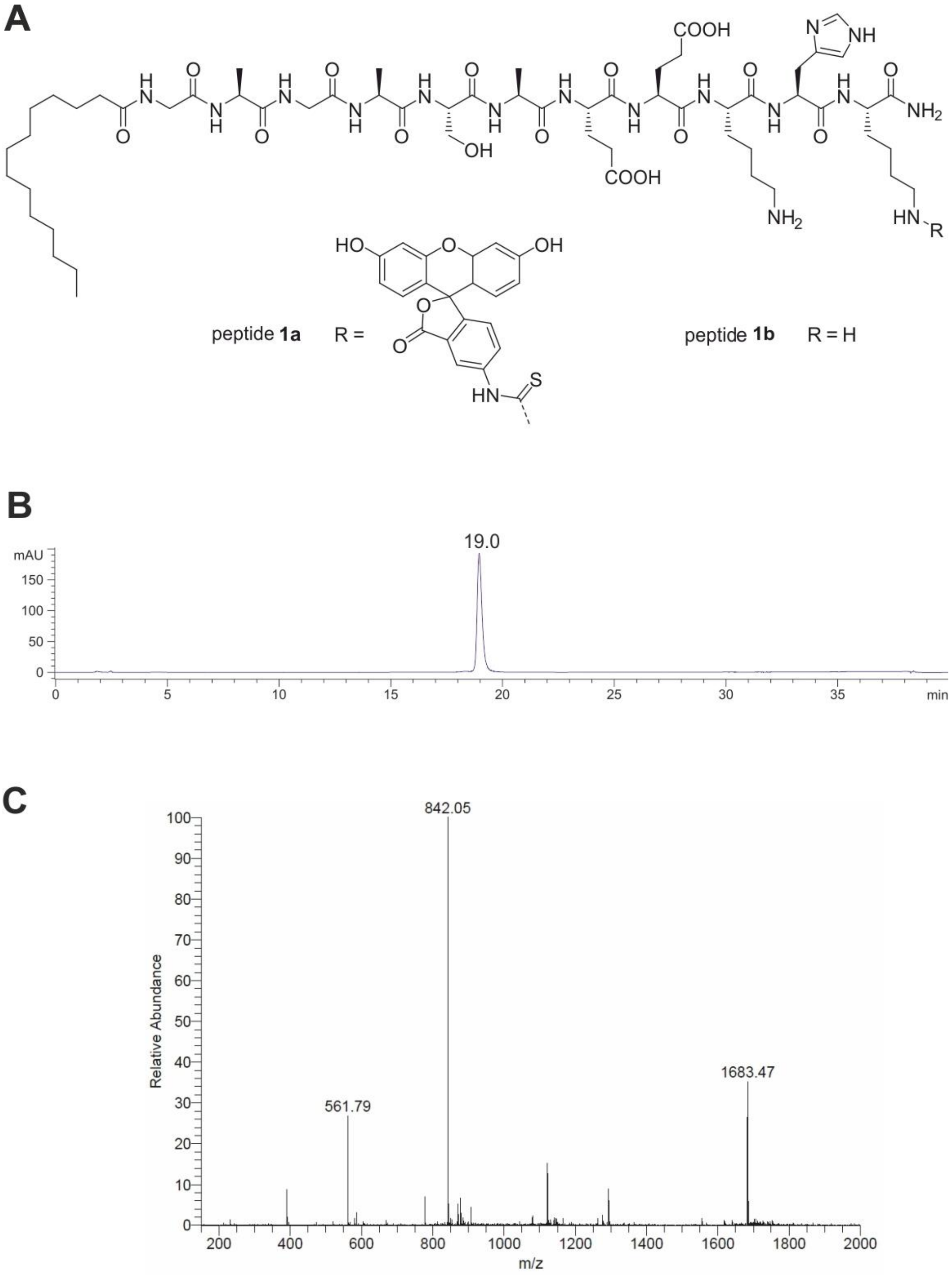

Supporting Figure S9. (**A**) Chemical structure of peptide **1a** and **1b**. (**B**) HPLC chromatogram of peptide **1a** (gradient: 10-90% MeCN + 0.1% TFA in 30 min). (**C**) ESI MS-spectrum of peptide **1a** (MF: $C_{79}H_{113}N_{17}O_{22}S$).

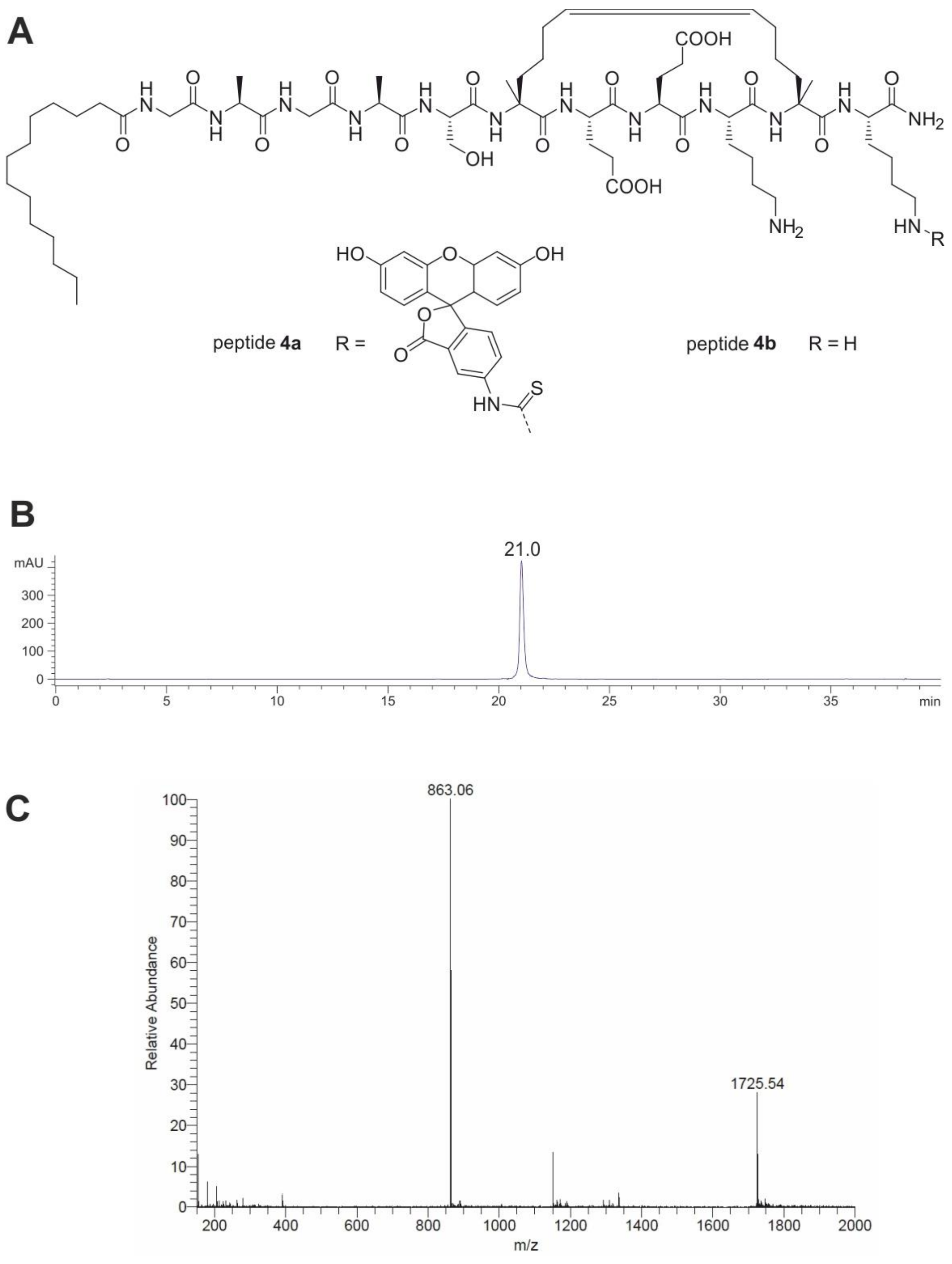

Supporting Figure S10. (**A**) Chemical structure of peptide **4a** and **4b**. (**B**) HPLC chromatogram of peptide **4a** (gradient: 10-90% MeCN + 0.1% TFA in 30 min). (**C**) ESI MS-spectrum of peptide **4a** (MF: $C_{84}H_{123}N_{15}O_{22}S$).

4 Supporting Tables

Supporting Table S1. Detailed overview of all synthesized peptides based on the non-modified Gnat1 sequence

Entry	*N*-Term mod. [a]	Sequence	Purity [%] [b]	HPLC (t_R, [min]) [c]	Yield [%] [d]	HRMS Calc.	HRMS (found)
**1a**	**F**	Myr – G A G A S A E E K H K	99	19.0	9	841.89779	841.89816 $[M+2H]^{2+}$
**1b**	**n.m.**		98	14.4	10	1293.75251	1293.75635 $[M+H]^{+}$
**2**	**F**	Myr – G A $\boldsymbol{R_5}$ A S $\boldsymbol{S_5}$ E E K H K	99	21.3	8	1804.89786	1804.89759 $[M+H]^{+}$
**3**	**F**	Myr – G $\boldsymbol{R_5}$ G A $\boldsymbol{S_5}$ A E E K H K	99	21.3	11	1774.88729	1774.88848 $[M+H]^{+}$
**4a**	**F**	Myr – G A G A S $\boldsymbol{S_5}$ E E K $\boldsymbol{S_5}$ K	98	21.0	10	862.93384	862.93349 $[M+2H]^{2+}$
**4b**	**n.m.**		99	15.8	14	1335.82461	1335.82915 $[M+H]^{+}$
**5**	**F**	Myr – G A G A S A $\boldsymbol{R_5}$ E K $\boldsymbol{S_5}$ K	97	23.1	8	833.93110	833.93099 $[M+2H]^{2+}$
**6**	**F**	Myr – G A G A S $\boldsymbol{R_5}$ E E $\boldsymbol{S_5}$ H K	98	22.1	9	867.41582	867.41581 $[M+2H]^{2+}$
**7**	**F**	Lau – G A G A S $\boldsymbol{S_5}$ E E K $\boldsymbol{S_5}$ K	99	19.2	9	848.91819	848.91835 $[M+2H]^{2+}$
**8**	**F**	Lau – G A G A S A $\boldsymbol{R_5}$ E K $\boldsymbol{S_5}$ K	98	19.9	7	819.91545	819.91577 $[M+2H]^{2+}$
**9**	**F**	Lau – G A G A S $\boldsymbol{R_5}$ E E $\boldsymbol{S_5}$ H K	98	19.2	8	853.40017	853.40034 $[M+2H]^{2+}$
**10a**	**F**	Ac – G A G A S $\boldsymbol{S_5}$ E E K $\boldsymbol{S_5}$ K	98	10.9	11	1556.67261	1556.67615 $[M+H]^{+}$
**10b**	**n.m.**		98	7.2	12	1167.63681	1167.64016 $[M+H]^{+}$

[a] F = fluorescein, n.m. = no modification, Ac= acetylation
[b] Calculated from UV-absorbance at 210 nm
[c] Retention time of purified peptides by analytical HPLC (10-90% MeCN (0.1% TFA), 30 min)
[d] Yield was determined according vendor loading

Supporting Table S2. DNA content values of the flow cytometry measurements for L cells and HeLa cells. Triplicate measurements, error = 1σ. Nocodazole was used at the maximum tolerated concentration of 1 µM.

Peptide	Conc.	DNA-content 4N	
		L cells	HeLa cells
**DMSO**	1%	14.7 ± 0.4	13.3 ± 2.9
**4**	1 µM	23.0 ± 3.5	18.6 ± 6.0
	5 µM	27.5 ± 0.6	27.2 ± 4.3
	10 µM	33.0 ± 4.6	24.0 ± 2.5
	25 µM	38.3 ± 6.7	31.0 ± 3.8
	50 µM	42.4 ± 8.5	35.8 ± 5.1
**1**	50 µM	28.0 ± 0.9	24.4 ± 2.3
**10**	50 µM	15.5 ± 3.9	14.7 ± 3.8
nocodazole	1 µM	75.4 ± 15.0	68.6 ±4.2

Supporting Table S3. (**A**) Empirical formula and structures of internal standards clozapine and S-Adenosyl Homocysteine (SAH) with the corresponding MRM transitions (**B**) MS settings for peptide and standard quantification.

A

**Clozapine**	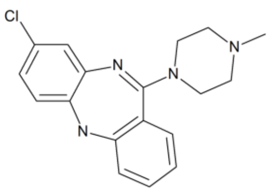	Molecular weight: 326.82<br>Molecular formula:<br>$C_{18}H_{19}ClN_{4}$
**SAH**	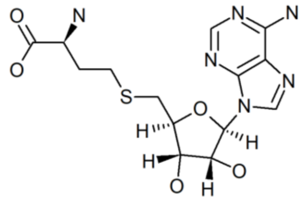	Molecular weight: 384.41<br>Molecular formula:<br>$C_{14}H_{2}0N_{6}O_{5}S$

B

Compound	Mode	Parent ion	Daughter ion	Dwell	Cone Voltage	Collision Energy
**10**	ESI +ve	585	171	0.163s	30	40
**4**	ESI +ve	669	129	0.163s	30	40
**R8**	ESI +ve	328	70	0.163s	30	35
**Clozapine**	ESI +ve	327	270	0.163s	30	35
**SAH**	ESI +ve	385	136	0.163s	30	15

Supporting Table S4. (**A**) Total drug content in MDA-MC-231 cells on incubation of 25 µM compound for 0.5 h and (**B**) Media concentrations post incubation.

A

Compound	Measured Conc (nM)	Dilution Factor	# of cells (million)	pMols/million cells
**10**	0.555	10	11.5	0.241
**4**	155	10	13.0	59.9
**R8**	108	10	10.4	51.7

B

Compound	Media Conc post incubation (µM)
**10**	15.8
**4**	11.7
**R8**	3.86

Supporting Table S5. (**A**) Sub-cellular drug content in MDA-MC-231 cells on incubation of 25 µM compound 0.5hrs / 72 h (LLOQ: lower limit of quantification), (**B**) media concentrations pre and post incubation.

A

Compound	Sub-cellular compartment	Incubation time (hrs)	Measured Conc (nM)	Dilution Factor	# of cells (million)	pMols/million cells
**4**	Cytoplasm	0.5	<LLOQ	2	18.2	<LLOQ
		72	<LLOQ	2	23.4	<LLOQ
	Nuclear	0.5	<LLOQ	2	18.2	<LLOQ
		72	<LLOQ	2	23.4	<LLOQ
**10**	Cytoplasm	0.5	<LLOQ	2	16.8	<LLOQ
		72	0.491	2	19.9	0.00987
	Nuclear	0.5	3.67	10	16.8	0.219
		72	8.98	10	19.9	0.451
**R8**	Cytoplasm	0.5	3.59	10	12.3	0.584
		72	2.92	10	8.98	0.651
	Nuclear	0.5	6.91	10	12.3	0.563
		72	5.73	10	8.98	0.638

B

Compound	Media Conc pre incubation (µM)	Media Conc post incubation (µM)
**10**	34.0	33.0
**4**	25.5	32.4
**R8**	15.5	Not measured

Supporting Table S6. Representative r^2 and QC errors from a single sample batch.

	r^2	QC 500nM n=2	QC 100nM n=2
**10**	0.996	3.6%	8.55%
**4**	0.995	11.1%	1.95%
**R8**	0.996	4.85%	13.7%

Supporting Table S7. Lower limit of quantification (LLOQ) for the different compartments and peptides.

	MS LLOQ / pmol per 10^6 cells				
	**total cell**	**nucleus**		**cytosol**	
**peptide**	0.5 h	0.5 h	72 h	0.5 h	72 h
**4**	0.004	0.001	0.001	0.001	0.001
**10**	0.04	0.04	0.04	0.04	0.04
**R8**	0..2	0.03	0.64	0.03	0.03

5 References

[1] H. Motulsky, A. Christopoulos, *Fitting models to biological data using linear and nonlinear regression*. *A practical guide to curve fitting*, Oxford University Press, Oxford, New York, **2004**.

[2] a) M. Jaiswal, E. K. Fansa, S. K. Kosling, T. Mejuch, H. Waldmann, A. Wittinghofer, *J. Biol. Chem.* **2016**, *291*, 20766–20778; b) S. Veltel, A. Kravchenko, S. Ismail, A. Wittinghofer, *FEBS Lett.* **2008**, *582*, 2501–2507.

[3] a) A Mateus et al. *Mol. Pharmaceutics*, **2013**, *10*, 2467–2478; b) L.J. Gordan et al. *J. Biomol. Screen.* **2016**, *21*, 156–164; c) X. Zhang et al. *Toxicol. In Vitro*, **2015**, *29*, 251–258; d) K. Teuscher, et al. *J. Med. Chem.*, **2017**, *60*, 157–169.
